# Dietary diversity and healthy life expectancy among Chinese older adults: a multistate analysis of CLHLS, 2008–2018

**DOI:** 10.3389/fpubh.2026.1922052

**Published:** 2026-10-05

**Authors:** Jiaqi Shi, Shimin Su, Yumeng Zhang, Tingting Xu, Huiling Dong, Bingyi Wu, Zhenhua Feng

**Affiliations:** 1 School of Management, Shandong Second Medical University, Weifang, China; 2 College of Liberal Arts and Social Sciences, City University of Hong Kong, Hong Kong, Hong Kong SAR, China; 3 School of Psychology, Shandong Second Medical University, Weifang, China; 4 School of Nursing, Shandong Second Medical University, Weifang, China; 5 School of Marxism, China University of Petroleum, East China, Qingdao, China

**Keywords:** dietary diversity, healthy life expectancy, life expectancy, microsimulation, older adults

## Abstract

**Objective:**

This study estimated healthy life expectancy (HLE) among Chinese older adults across varying levels of dietary diversity, and examined sex and urban-rural differences.

**Methods:**

Drawing on the Chinese Longitudinal Healthy Longevity Survey (CLHLS) from 2008 to 2018, data on 9,953 older adults aged 65 years and older were included. Transition probabilities among health statuses were estimated. Further, a microsimulation model was used to calculate life expectancy (LE), HLE, and HLE/LE for older adults with varying levels of dietary diversity. Sex and urban-rural differences were also examined descriptively. *Z*-tests were applied to determine whether differences in HLE within subgroups were statistically significant (*p* < 0.05).

**Results:**

Compared with older adults with low dietary diversity, those with high dietary diversity exhibited a higher probability of transitioning from health to disability. However, they also exhibited a greater likelihood of recovering from disability and returning to health. Additionally, older adults with high dietary diversity exhibited lower transition probabilities to death from both health and disability. Across all age groups, older adults with high dietary diversity had higher LE and HLE values, albeit with lower HLE/LE ratios. At age 65, the difference in LE between the low- and high-dietary-diversity groups was 1.26 years [14.54 years (95% CI: 14.42–15.34) versus 15.80 years (95% CI: 15.55–16.17)]. Meanwhile, the corresponding difference in HLE was 0.90 years [13.32 years (95% CI: 13.25–13.95) versus 14.22 years (95% CI: 13.91–14.58)]. Stratified analyses further indicated that differences in HLE between dietary diversity groups were consistently observed across sex and urban-rural residence groups. Descriptively, the differences appeared larger among men and urban residents.

**Conclusion:**

Higher dietary diversity was associated with a higher probability of transition from disability to health, lower probabilities of transition from either health or disability to death, and higher LE and HLE. These associations were observed across sex and urban-rural residence groups. Descriptively, larger differences in HLE between dietary diversity groups were observed among males and urban residents.

## 1 Introduction

The importance of diet as a key modifiable determinant of health becomes increasingly evident with advancing age (1). Age-related declines in digestive, neurological, and other physiological functions (2, 3), coupled with lower socioeconomic status and impaired functional capacity (4, 5), collectively increase nutritional vulnerability and health risks. With an increasing older adult population, China faces a substantial challenge in achieving healthy aging. For instance, the China Report on Nutrition and Health of the Elderly reported that 48.4% of older adults in China are at risk of malnutrition (6).

Dietary diversity is a key dimension of dietary assessment, and is closely associated with nutritional adequacy. Rather than relying on a single food source, it emphasizes the intake of a variety of foods over a specific period, thereby reflecting the overall balance of an individual’s diet (7, 8). Evidence suggests that greater dietary diversity contributes to a more adequate nutrient profile, supports physical function maintenance, and protects against a decline in multidimensional functional outcomes related to healthy aging (9). Specifically, greater dietary diversity has been associated with a lower risk of the onset and progression of age-related diseases (10–12). Further, it is closely associated with reduced risks of diabetes (13), cardiovascular disease (14), and cataract (15). It has also been linked to reduced risks of cognitive impairment (16) and mortality (17) in older adults, and improved memory function (18). Moreover, it plays a critical role in preventing frailty (19). Conversely, insufficient dietary diversity may result in inadequate nutrient intake and reduced energy reserves. This can not only impair immune function (20) but also increase the risks of infectious diseases (21) and depression (22).

However, studies on dietary diversity and health outcomes in older adults are limited by small sample sizes, short follow-up periods, and cross-sectional designs. This limits their ability to capture long-term associations. Moreover, most studies have focused on disease incidence rather than functional health. For older adults, promoting health and preserving function may be more meaningful than merely preventing or treating diseases. Additionally, dietary modification represents a low-cost strategy to maintain functional capacity throughout life (23). The concept of healthy life expectancy (HLE) emphasizes not only longevity but also quality of life without functional limitations (24), shifting interventions from “treating disease” to “preserving function.” Therefore, accurately assessing the association between dietary diversity and HLE is essential for informing strategies related to healthy aging. To this end, we used four-wave Chinese Longitudinal Healthy Longevity Survey (CLHLS) data (2008–2018) with microsimulation and Monte Carlo simulation to estimate life expectancy (LE), HLE, and the HLE/LE ratio by dietary diversity level, and to examine sex and urban-rural differences. Our findings may contribute to the evidence base on dietary diversity and healthy longevity amid rapid population aging. Our findings aim to inform dietary-based strategies for promoting healthy longevity amid rapid population aging.

## 2 Methods

### 2.1 Data

The data were obtained from the CLHLS database. The survey was conducted by the Center for Healthy Aging and Development Studies at Peking University, and included participants from 23 provinces (autonomous regions and municipalities directly under the central government) in China. The data collection process strictly followed the ethical norms of scientific research and was approved by the Institutional Review Board of the Peking University Health Science Center (approval number: IRB00001052-13074). We extracted four waves of panel data (2008, 2011, 2014, and 2018) from the CLHLS database. According to the inclusion and exclusion criteria shown in Figure 1, 9,953 subjects were included in this study.

**Figure 1:**
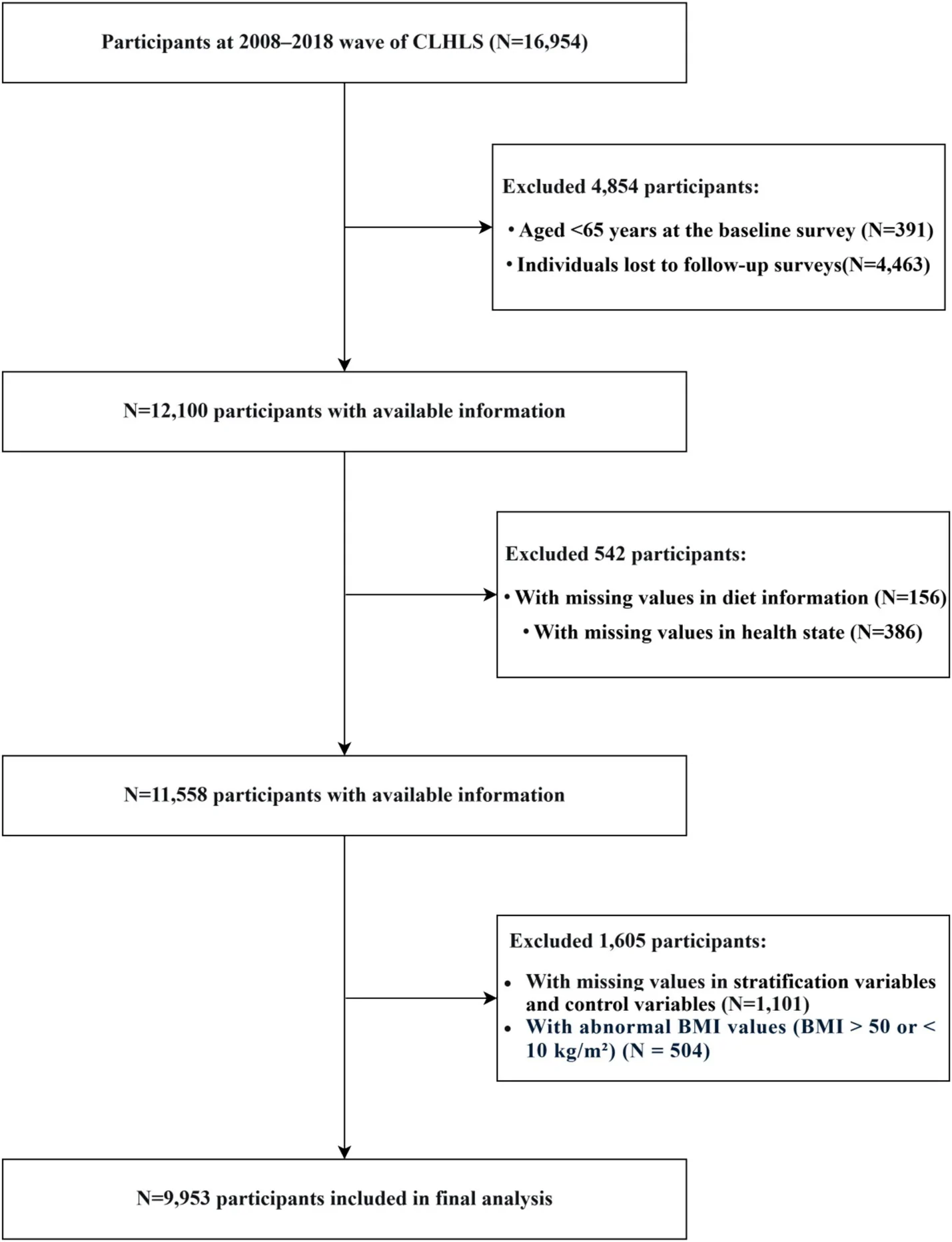
Flowchart of participant selection.

### 2.2 Variables

#### 2.2.1 Definition of health states

Activities of daily living were treated as a time-varying variable to assess participants’ health states and were measured using the six-item Katz scale. This scale is a well-established and reliable assessment tool covering eating, dressing, transferring, toileting, bathing, and continence (25). Each item originally had three ordered response categories: “independent,” “requires some assistance,” and “unable to perform.” In this study, the latter two categories were combined into “unable to perform.” Participants who could independently perform all six activities were classified as having no activity limitations (health state), whereas those unable to independently perform at least one activity were classified as having activity limitations (disability state); death was defined as the absorbing state. This classification is consistent with established approaches used in multistate life tables (MSLT) and the Sullivan method for estimating health expectancy (26). However, this approach may result in some loss of information regarding the severity gradient of disability, preventing further differentiation among mild, moderate, and severe disability levels.

#### 2.2.2 Assessment of independent variable

Dietary diversity was treated as a time-varying variable from 2008 to 2018. It was assessed at each survey wave using the Dietary Diversity Score (DDS). This score was calculated based on the consumption frequency of 13 food items in the CLHLS questionnaire: fresh fruits, fresh vegetables, meat, fish, eggs, soy products, preserved vegetables, sugar, garlic, dairy products, nut products, mushrooms or algae, and tea. The consumption frequency of the first two items (fresh fruit and fresh vegetables) was measured using four response options: daily, often, occasionally, and seldom or almost never. Respondents who answered “daily” or “often” received 1 point, while those who answered “occasionally” or “seldom or almost never” received 0 points. For the remaining 11 food items, the consumption frequency was measured using five response options: daily, weekly, monthly, occasionally, and seldom or almost never. Respondents who answered “daily” or “weekly” received 1 point, while those who answered “monthly,” “occasionally,” or “seldom or almost never” received 0 points. The scores for all food items were summed to obtain each respondent’s DDS, with scores ranging from 0 to 13 and higher scores indicating greater dietary diversity. Following extant studies (27), a score below 7 was classified as low dietary diversity, and a score of 7 or higher was classified as high dietary diversity.

#### 2.2.3 Assessment of stratification variables and control variables

Stratification variables included sex and residence. Residence was derived from the question on the respondent’s current place of residence, with cities and towns merged into the “urban” category.

The control variables included marital status, educational attainment, smoking, drinking, and BMI. Marital status as married and single; educational attainment as formal education and no formal education; smoking, drinking were each dichotomized as “yes” or “no” based on current behavioral status; and BMI was classified as underweight (<18.5 kg/m^2^), normal weight (18.5–23.9 kg/m^2^), and overweight (≥24 kg/m^2^). Except for sex and educational attainment, all other variables (residence, marital status, smoking, drinking, and BMI) were treated as time-varying variables, updated at each wave based on the respondent’s status at that time point.

### 2.3 Statistical analysis

A discrete-time MSLT model was used to estimate HLE. Specifically, stochastic microsimulations were conducted using the Stochastic Population Analysis for Complex Events (SPACE) program (28). This program is based on a first-order Markov chain and allows individual-level sampling weights to be incorporated into the microsimulation process, thereby accounting for the sampling design and improving the representativeness of the simulated population (29). Under the first-order Markov assumption, the probability of transitioning to a future health state depends on an individual’s current health status but is independent of their previous health history. This assumption is widely used in state-transition modeling for health, economic, and demographic projections (30, 31). Given the discrete multiyear follow-up intervals of the CLHLS dataset, the first-order Markov approach was considered appropriate for modeling longitudinal health transitions while maintaining model parsimony. Compared with higher-order models, it also reduces the risk of overfitting and avoids sparse transition probabilities that may arise when estimating more complex transition structures from limited longitudinal observations (32). The analytical procedures were as follows:

1. The intervals between consecutive survey waves were discretized into 1 year cycles. For individuals with a change in their health states between two consecutive waves, the timing of the transition was randomly allocated to one of the intervening annual cycles. For instance, when the interviews were conducted in 2014 and 2018, the transition was randomly assigned to 2015, 2016, or 2017, each with a probability of 1/3. Conversely, when the health state remained identical across consecutive interviews, no transition was assumed during the interval.
2. Considering two living health states, health (state 1) and disability (state 2), and one absorbing health state, death (state 3), all possible transitions across health states over time include the following: staying in the same health state (state 1 to 1 and 2 to 2), worsening health (state 1 to 2), improving health (state 2 to 1), and mortality (state 1/2 to 3; Figure 2).
3. Multinomial transition models were used to estimate the probabilities of moving between the health states over each observation interval. For each individual, a transition record was constructed using the health state observed at time *t* and the health state observed at the subsequent assessment *t* + 1. The dependent variable was the health state at *t* + 1. The possible outcomes included remaining in the current state, transitioning to another living health state, and transitioning to death. Death was treated as an absorbing state. For individuals in the current health state *r*, the probability of transitioning to state *s* at the subsequent assessment was modeled as follows:

$$P_{\mathrm{it}}(s|r)=P(H_{i,t+1}=s|H_{i,t}=r,X_{\mathrm{it}})$$

where *H_i, t_* denotes the health state of individual *i* at time *t*; *H_i, t + 1_* denotes the subsequent health state; and *X* represents the covariates included in the transition model. These covariates included DDS, age, and other pre-specified individual characteristics.

**Figure 2:**
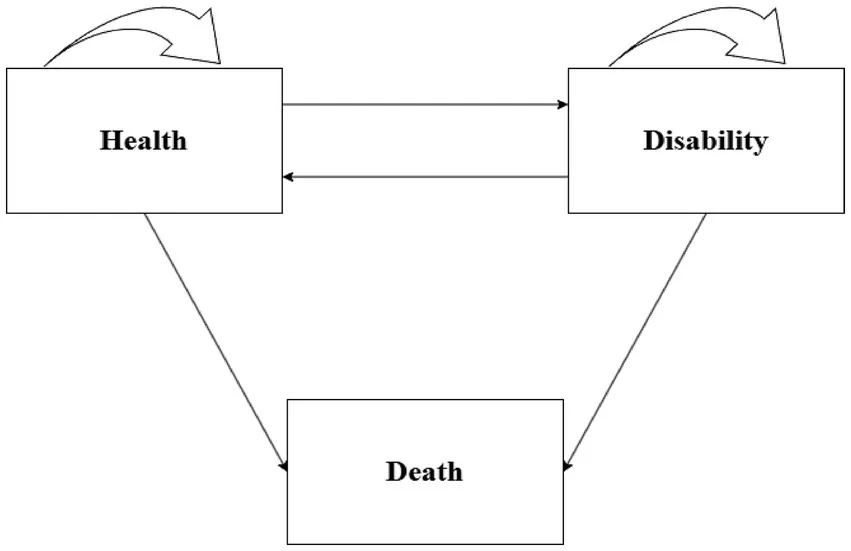
States and possible transitions across states.

For each current health state, a separate multinomial logistic regression model was estimated with one subsequent health state designated as the reference category. The model estimated the relative probability of transitioning to each of the other possible states. The predicted probabilities were obtained using the multinomial logistic transformation:


$$P_{\mathrm{it}}(s|r)=\frac{\exp(X_{\mathrm{it}}^{⊤}\beta_{\mathrm{rs}})}{\sum_{k=1}^{K}\exp(X_{\mathrm{it}}^{⊤}\beta_{\mathrm{rk}})}$$

where *K* is the number of possible health states and *β_rs_* denotes the coefficient vector for the transition from current state *r* to subsequent state *s*. The resulting probabilities were constrained to lie between 0 and 1, and sum to one across all possible subsequent states.

1. The transition probabilities described above were incorporated into a Monte Carlo–based stochastic microsimulation model to estimate LE and HLE. A discrete-time Markov chain algorithm based on 1-year transition probabilities was used to update each simulated individual’s health state during each annual cycle according to age, DDS level, and other model covariates. An initial cohort of 100,000 individuals aged 65 years was generated from the initial state matrix and individual health trajectories were simulated annually until death or 100 years of age. An event history recorder accumulated the person-years lived in healthy and disabled states, which were used to estimate the LE and HLE, respectively. The estimates were stratified by sex, residence, and DDS level, and 30 bootstrap replicates were performed to derive empirical 95% confidence intervals.

#### 2.3.1 *Z*-test

To compare whether significant differences existed in HLE at the same age among older adults with different characteristics, the *Z*-test was used. The *Z*-test statistic was calculated as follows:


$$Z=\frac{\mathrm{HL}E_{1}-\mathrm{HL}E_{2}}{\sqrt{SE_{1}^{2}+SE_{2}^{2}}}$$

## 3 Results

### 3.1 Baseline characteristics of the study population

This study included longitudinal data from 2008 to 2018, with 2008 as the baseline. The baseline sample comprised 9,953 older adults. Regarding age composition, the largest proportion of participants was aged 85–94 years (33.80%), followed by those aged ≥ 95 years (33.68%), 75–84 years (18.54%), and 65–74 years (13.99%). Regarding residence, 65.76% of participants were rural residents. Regarding baseline health status, 75.41% of participants were classified as healthy, with the remainder in the disabled state.

### 3.2 Health state transition probabilities by dietary diversity

Overall, the probability of transitioning from health to disability increased with age, whereas the probability of recovering from disability to health decreased with age. Older adults in the high dietary diversity group consistently exhibited a higher probability of transitioning from health to disability than those in the low dietary diversity group, and this between-group difference widened with age. Conversely, the probability of transition from disability to health was lower among older adults in the low dietary diversity group than among those in the high dietary diversity group, although this difference narrowed with age (Figure 3). Furthermore, the probabilities of transitioning from health to death and from disability to death increased with age. Older adults in the low dietary diversity group consistently exhibited higher probabilities of transitioning from health to death and from disability to death than those in the high dietary diversity group, with the between-group differences widening progressively with age (Figure 4). At age 65, the transition probabilities from health to disability, disability to health, health to death, and disability to death were 0.010 (95% CI: 0.008–0.012), 0.189 (95% CI: 0.172–0.249), 0.024 (95% CI: 0.021–0.026), and 0.080 (95% CI: 0.072–0.107), respectively, among older adults with low dietary diversity. The corresponding probabilities among those with high dietary diversity were 0.011 (95% CI: 0.008–0.012), 0.211 (95% CI: 0.208–0.302), 0.021 (95% CI: 0.020–0.023), and 0.061 (95% CI: 0.046–0.069), respectively (see Table 1).

**Figure 3:**
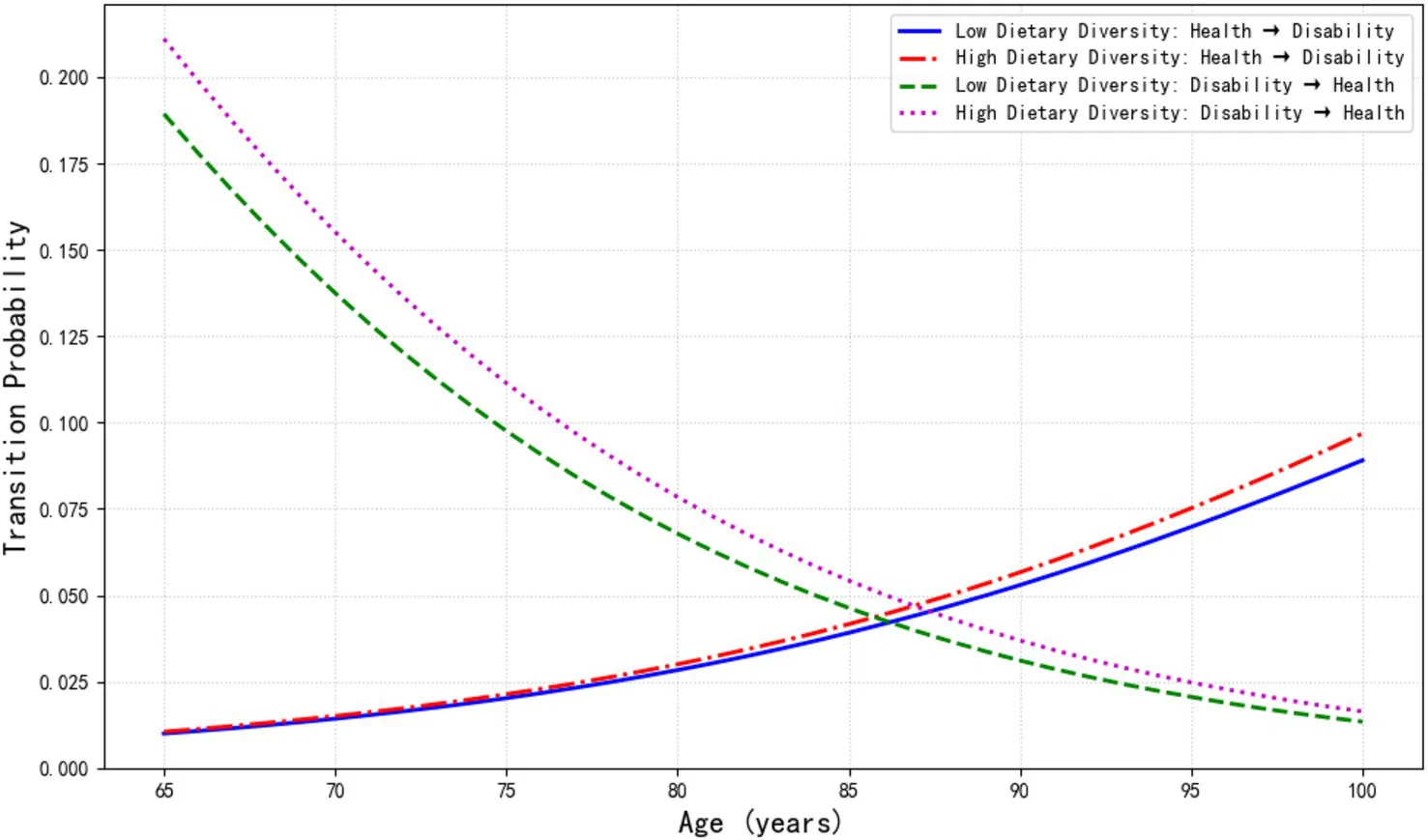
Transition probabilities between health and disability by dietary diversity.

**Figure 4:**
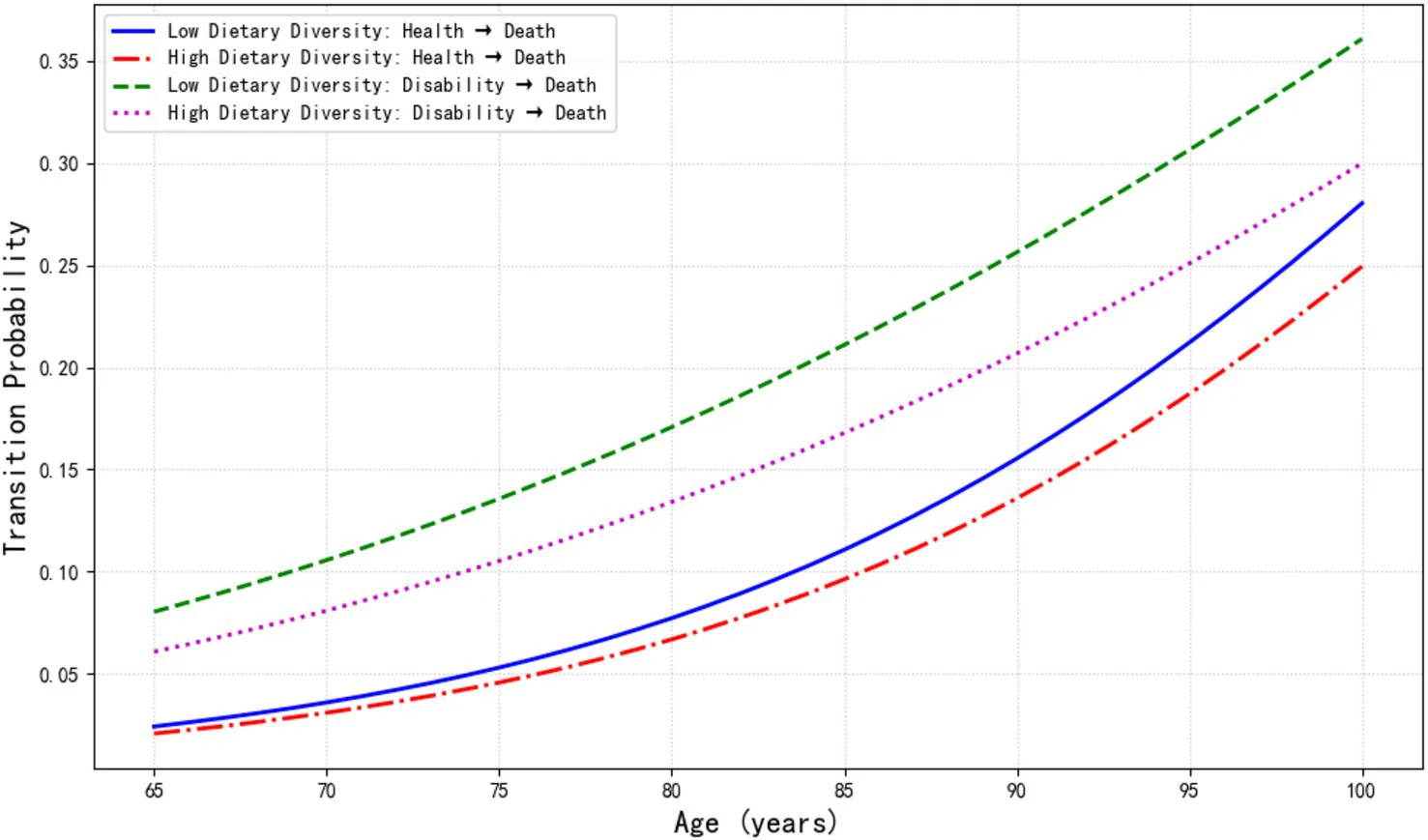
Transition probabilities from health and disability to death by dietary diversity.

**Table 1:** Baseline characteristics.

Variables	2008 Year		2011 Year		2014 Year		2018 Year	
	*N*	Proportion (%)	*N*	Proportion (%)	*N*	Proportion (%)	*N*	Proportion (%)
Age								
65–74	1,392	13.99	820	18.38	408	16.14	21	1.39
75–84	1845	18.54	1,210	27.12	951	37.62	832	55.17
85–94	3,364	33.80	1,393	31.22	752	29.75	491	32.56
95 and above	3,352	33.68	1,039	23.29	415	16.42	162	10.74
Sex								
Male	4,245	42.65	2,111	47.31	1,227	48.54	725	48.08
Female	5,708	57.35	2,351	52.69	1,299	51.38	781	51.79
Residence								
Urban	3,408	34.24	2,271	50.90	1,345	53.20	879	58.29
Rural	6,545	65.76	2,191	49.10	1,181	46.72	627	41.58
Education								
No formal education	6,586	66.17	2,575	57.71	1,312	56.57	694	46.02
Formal education	3,367	33.83	1887	42.29	1,214	43.35	812	53.85
Marital status								
Married	2,447	24.59	1,671	37.45	1,096	43.35	669	44.36
Single	7,506	75.41	2,791	62.55	1,430	56.57	837	55.50
Smoke								
Yes	1714	17.22	828	18.56	455	18.00	253	16.78
No	8,239	82.78	3,634	81.44	2071	81.92	1,253	83.09
Drink								
Yes	1728	17.36	787	17.64	412	16.30	230	15.25
No	8,225	82.64	3,675	82.36	2,114	83.62	1,276	84.62
BMI								
Underweight	3,705	37.22	1,211	27.14	472	18.67	238	15.78
Normal	5,041	50.64	2,342	52.49	1,382	54.67	785	52.06
Overweight	1,207	12.14	909	20.37	672	26.58	483	32.03
DDS								
Low	7,135	71.69	3,180	71.27	1794	70.97	1,046	69.36
High	2,818	28.31	1,282	28.73	732	28.96	460	30.50
Health states								
Health	7,506	75.41	3,366	33.82	2006	44.96	1,213	48.02
Disability	2,447	24.59	1,096	11.01	520	11.65	293	11.60
Death			5,491	55.17	1936	43.39	1,020	40.38

Urban-rural differences showed that at the same level of dietary diversity, urban older adults had a higher probability of transitioning from health to disability after age 85 than rural older adults. Meanwhile, the probability of transitioning from disability to health was higher among urban older adults before age 75. Moreover, urban older adults had lower probabilities of transitioning from both health and disability to death than rural older adults (Figures 5, 6). Sex differences showed that at the same level of dietary diversity, female older adults had a higher probability of transitioning from health to disability than male older adults. Among older adults with high dietary diversity, the probability of transitioning from disability to health was higher in females than in males before age 78 years; among those with low dietary diversity, this probability was higher in females than in males before age 86 years. In addition, among the low dietary diversity group and female older adults younger than 94 years in the high dietary diversity group, the probabilities of transitioning from both health and disability to death were lower than those among male older adults (Figures 7, 8). Descriptively, differences in health state transition probabilities between the dietary diversity groups appeared larger among male and urban older adults than among female and rural older adults.

**Figure 5:**
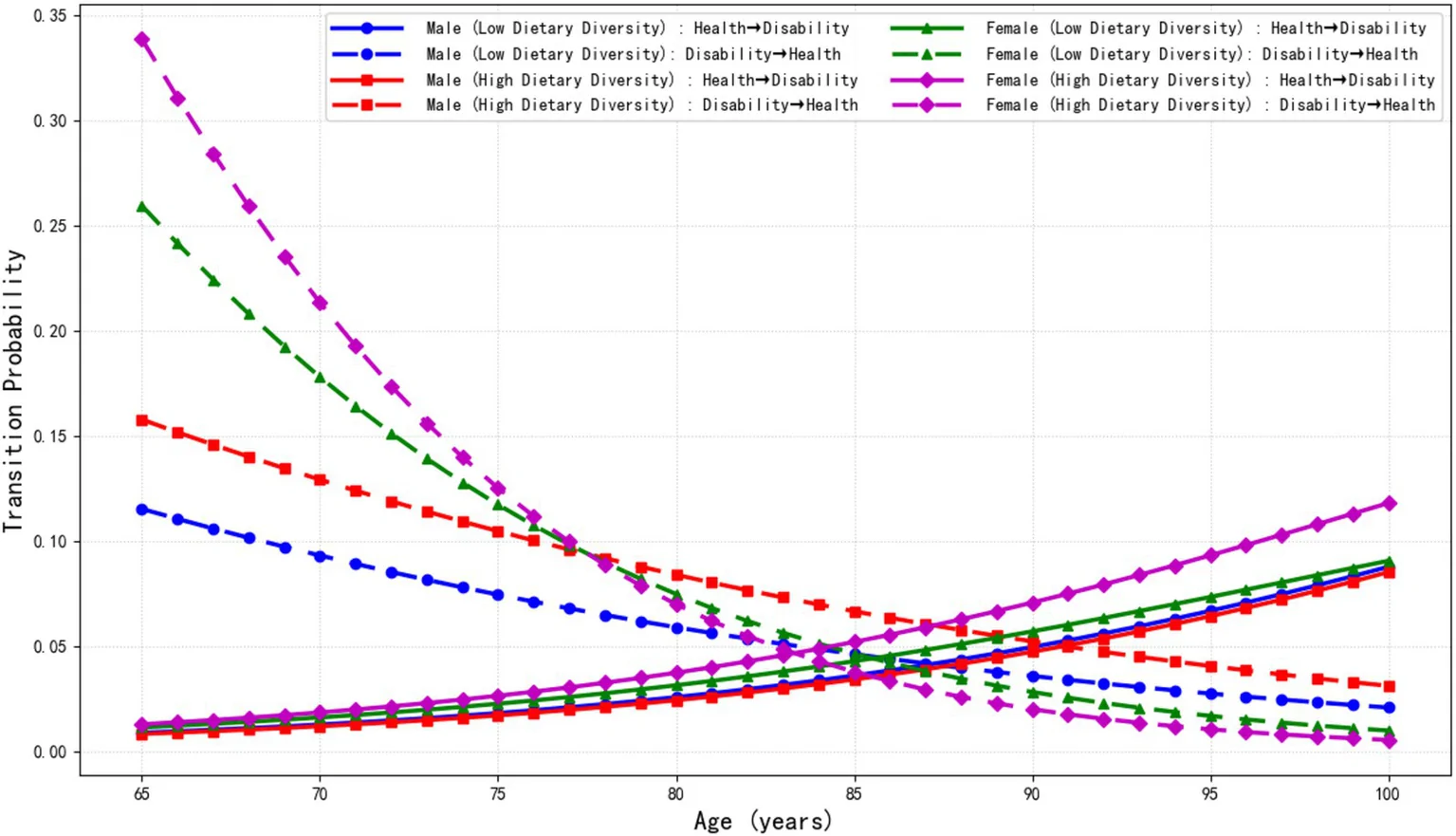
Transition probabilities between health and disability by dietary diversity and sex.

**Figure 6:**
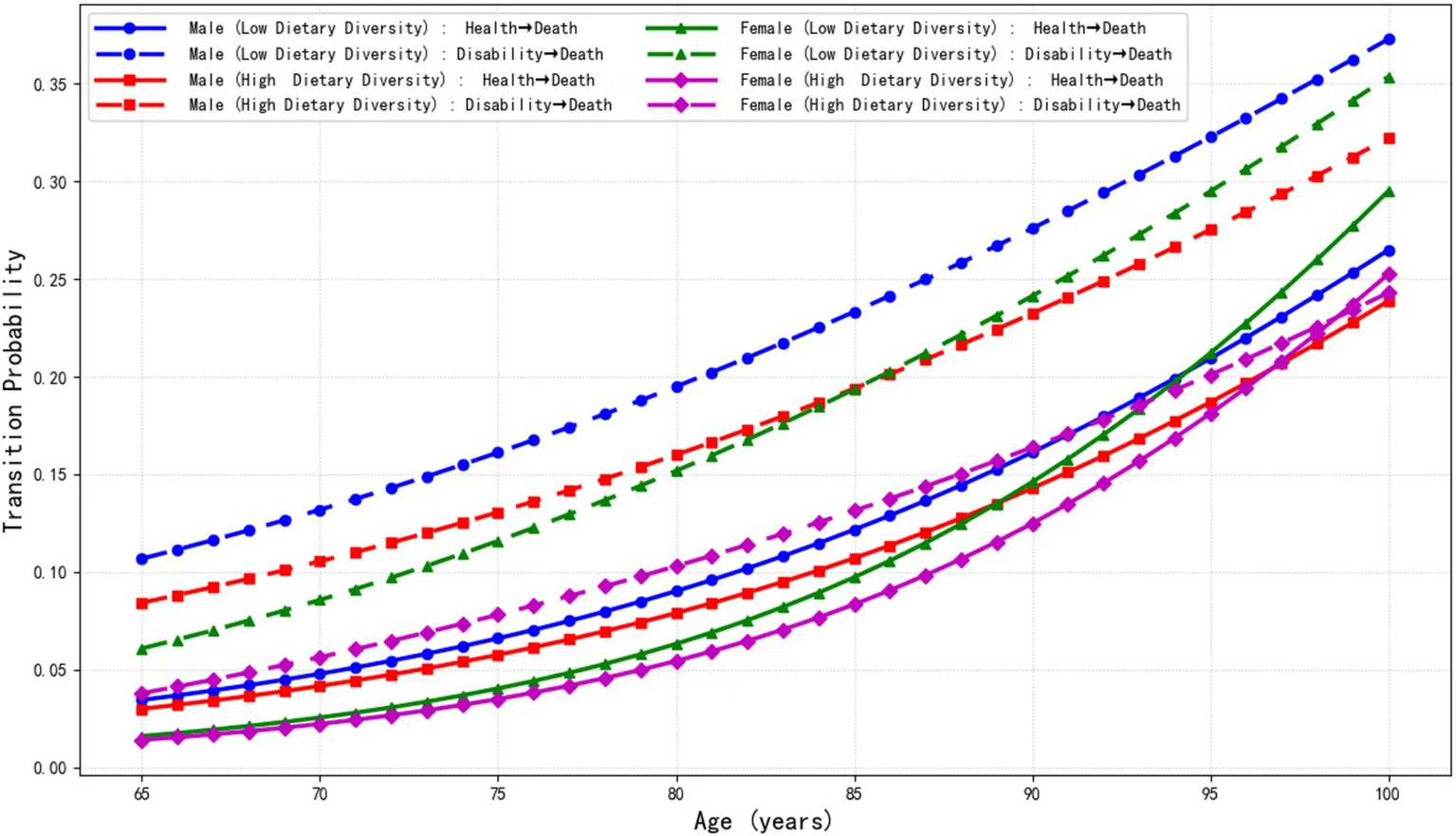
Transition probabilities from health and disability to death by dietary diversity and sex.

**Figure 7:**
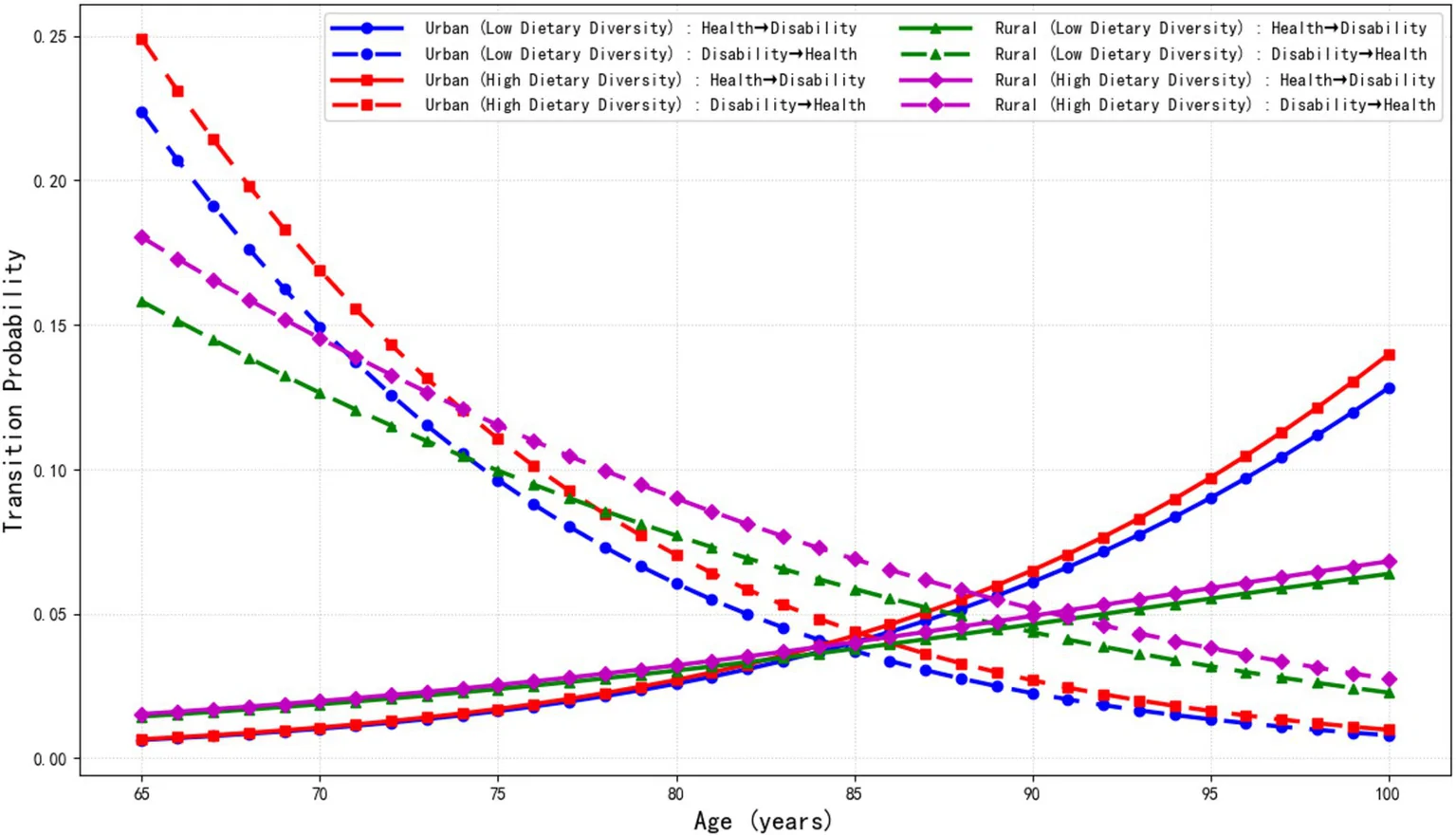
Transition probabilities between health and disability by dietary diversity and urban-rural residence.

**Figure 8:**
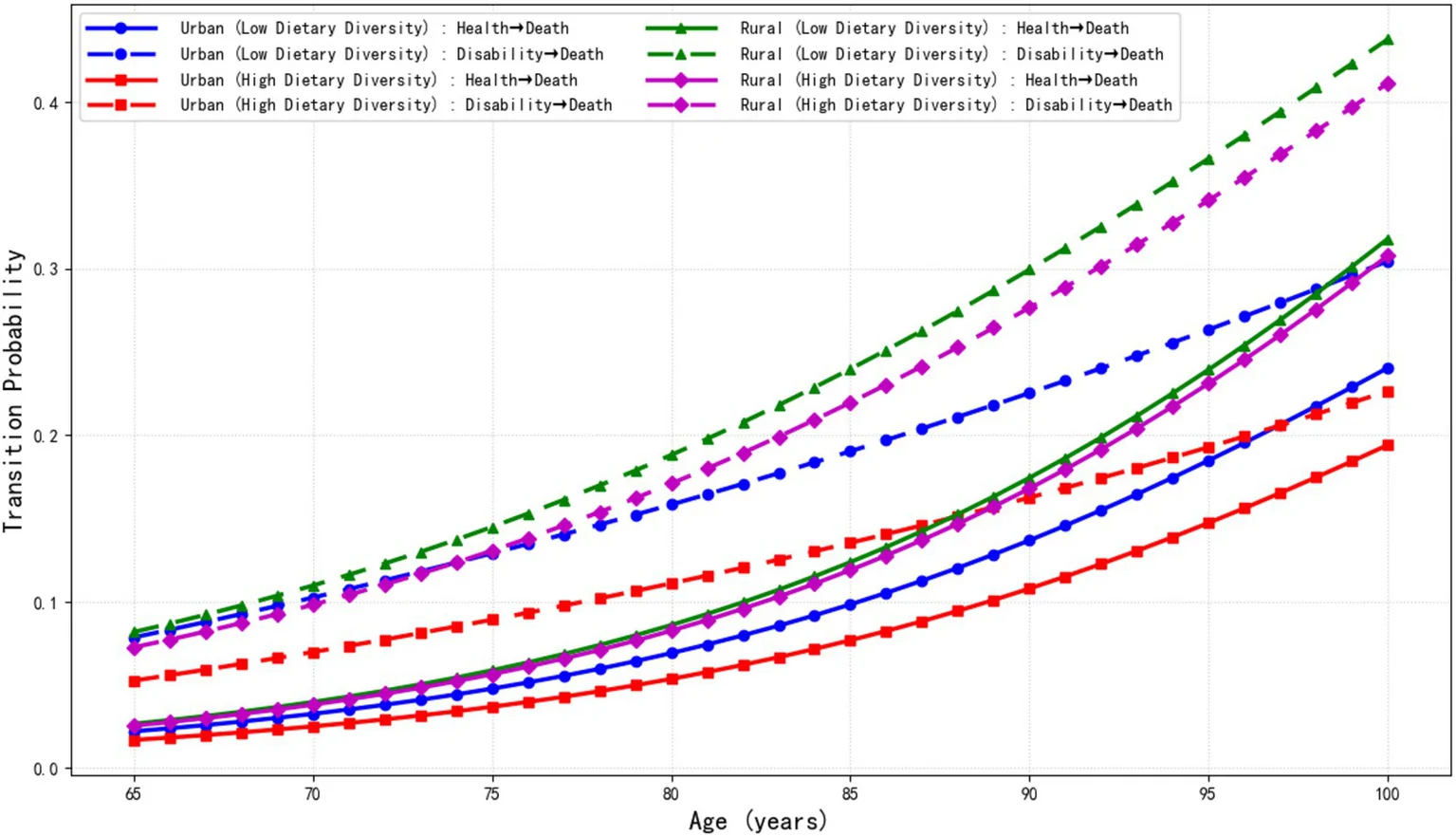
Transition probabilities from health and disability to death by dietary diversity and urban-rural residence.

### 3.3 LE, HLE, and HLE/LE by dietary diversity

As shown in Table 2, LE, HLE, and HLE/LE all declined with age. Older adults in the high dietary diversity group had higher LE and HLE values at each age. Meanwhile, their HLE/LE ratio was lower than that of those in the low dietary diversity group. For instance, at age 65, participants in the low dietary diversity group had an LE of 14.54 years (95% CI: 14.42–15.34), HLE of 13.32 years (95% CI: 13.25–13.95), and HLE/LE ratio of 91.56%, compared with 15.80 years (95% CI: 15.55–16.17), 14.22 years (95% CI: 13.91–14.58), and 89.97%, respectively, in the high dietary diversity group. *Z*-test results indicated that, among adults aged 65–90 years, HLE was higher in the high dietary diversity group than in the low dietary diversity group, and the corresponding differences were statistically significant (*p* < 0.05).

**Table 2:** LE, HLE, and HLE/LE by dietary diversity in older adults.

Age	Low dietary diversity			High dietary diversity			Z
	LE	HLE	(HLE/LE)%	LE	HLE	(HLE/LE)%	
65	14.54 (14.42–15.34)	13.32 (13.25–13.95)	91.56	15.80 (15.55–16.17)	14.22^***^ (13.91–14.58)	89.97	2.81
66	13.96 (13.83–14.72)	12.70 (12.67–13.33)	91.01	15.16 (14.85–15.54)	13.58^***^ (13.23–13.89)	89.53	2.93
67	13.30 (13.24–14.06)	12.07 (12.05–12.68)	90.70	14.55 (14.35–14.83)	12.96^***^ (12.70–13.28)	89.10	3.46
68	12.76 (12.72–13.41)	11.50 (11.51–12.03)	90.16	13.99 (13.74–14.24)	12.39^***^ (12.10–12.66)	88.55	3.57
69	12.26 (12.15–12.90)	11.00 (10.95–11.52)	89.69	13.38 (13.14–13.65)	11.79^***^ (11.49–12.04)	88.09	3.27
70	11.68 (11.59–12.36)	10.43 (10.41–10.97)	89.28	12.80 (12.61–13.09)	11.22^***^ (10.95–11.40)	87.60	3.83
71	11.15 (11.11–11.80)	9.90 (9.93–10.42)	88.80	12.25 (12.05–12.55)	10.66^***^ (10.38–10.88)	87.09	3.48
72	10.63 (10.59–11.29)	9.38 (9.41–9.89)	88.21	11.67 (11.58–11.98)	10.09^***^ (9.89–10.35)	86.43	3.75
73	10.16 (10.13–10.74)	8.91 (8.94–9.36)	87.68	11.17 (11.05–11.45)	9.56^***^ (9.37–9.77)	85.55	3.84
74	9.65 (9.64–10.27)	8.39 (8.45–8.89)	86.86	10.68 (10.56–10.97)	9.07^***^ (8.86–9.25)	84.96	4.16
75	9.23 (9.24–9.81)	7.97 (8.03–8.43)	86.33	10.21 (10.10–10.48)	8.59^***^ (8.42–8.77)	84.16	4.30
76	8.78 (8.79–9.32)	7.52 (7.58–7.94)	85.64	9.79 (9.62–10.03)	8.19^***^ (7.93–8.29)	83.59	4.47
77	8.37 (8.32–8.89)	7.11 (7.11–7.52)	84.91	9.26 (9.18–9.54)	7.64^***^ (7.46–7.81)	82.54	3.37
78	7.99 (8.00–8.46)	6.71 (6.74–7.08)	83.88	8.87 (8.68–9.11)	7.25^***^ (7.07–7.38)	81.67	4.10
79	7.58 (7.61–8.08)	6.29 (6.35–6.70)	83.00	8.47 (8.30–8.70)	6.84^***^ (6.64–6.96)	80.78	3.75
80	7.21 (7.22–7.63)	5.93 (5.96–6.27)	82.26	8.07 (7.87–8.32)	6.44^***^ (6.23–6.56)	79.84	3.53
81	6.84 (6.88–7.29)	5.55 (5.60–5.93)	81.09	7.65 (7.47–7.91)	6.03^***^ (5.85–6.16)	78.81	3.62
82	6.52 (6.56–6.92)	5.23 (5.22–5.57)	80.18	7.29 (7.10–7.55)	5.67^***^ (5.47–5.80)	77.68	3.13
83	6.22 (6.22–6.59)	4.91 (4.87–5.22)	78.93	6.93 (6.76–7.18)	5.29^***^ (5.10–5.43)	76.34	2.74
84	5.88 (5.90–6.24)	4.57 (4.55–4.88)	77.70	6.62 (6.40–6.83)	4.98^***^ (4.74–5.08)	75.15	3.02
85	5.60 (5.59–5.97)	4.29 (4.25–4.60)	76.55	6.31 (6.07–6.52)	4.66^***^ (4.43–4.76)	73.90	2.88
86	5.32 (5.30–5.64)	3.99 (3.96–4.28)	75.10	6.00 (5.76–6.21)	4.36^***^ (4.10–4.43)	72.65	2.88
87	5.04 (5.02–5.35)	3.72 (3.67–4.00)	73.73	5.70 (5.45–5.94)	4.05^**^ (3.80–4.17)	71.18	2.49
88	4.82 (4.75–5.11)	3.48 (3.39–3.74)	72.15	5.46 (5.19–5.69)	3.79^**^ (3.50–3.88)	69.52	2.25
89	4.60 (4.53–4.83)	3.25 (3.14–3.48)	70.58	5.18 (4.94–5.44)	3.52^**^ (3.24–3.62)	67.95	1.98
90	4.36 (4.28–4.61)	3.00 (2.89–3.24)	68.93	4.96 (4.71–5.20)	3.28^**^ (2.98–3.35)	66.17	2.02
91	4.17 (4.06–4.38)	2.79 (2.66–3.01)	67.04	4.71 (4.46–4.99)	3.04^*^ (2.75–3.13)	64.46	1.79
92	3.97 (3.87–4.17)	2.59 (2.45–2.78)	65.12	4.51 (4.25–4.79)	2.82 (2.51–2.91)	62.55	1.62
93	3.80 (3.67–4.00)	2.40 (2.24–2.60)	63.14	4.30 (4.05–4.61)	2.60 (2.30–2.68)	60.52	1.47
94	3.62 (3.49–3.85)	2.21 (2.05–2.39)	61.11	4.12 (3.86–4.43)	2.42 (2.10–2.48)	58.66	1.48
95	3.46 (3.33–3.68)	2.04 (1.88–2.22)	59.05	3.94 (3.67–4.28)	2.23 (1.92–2.29)	56.59	1.39
96	3.30 (3.16–3.53)	1.87 (1.72–2.04)	56.70	3.76 (3.51–4.14)	2.05 (1.75–2.10)	54.56	1.39
97	3.15 (3.02–3.39)	1.72 (1.56–1.87)	54.61	3.61 (3.35–3.97)	1.89 (1.59–1.93)	52.23	1.29
98	3.04 (2.89–3.27)	1.59 (1.42–1.73)	52.29	3.46 (3.21–3.86)	1.74 (1.44–1.78)	50.19	1.17
99	2.91 (2.78–3.15)	1.45 (1.29–1.59)	49.91	3.34 (3.08–3.75)	1.60 (1.30–1.64)	47.86	1.17
100	2.80 (2.66–3.05)	1.34 (1.16–1.46)	47.67	3.20 (2.95–3.65)	1.46 (1.18–1.50)	45.67	1.07

Values in parentheses are 95% confidence intervals (CIs). ^***^p < 0.001, ^**^p < 0.05, ^*^p < 0.1.

### 3.4 Sex differences in HLE by dietary diversity among older adults

As shown in Table 3, LE, HLE, and HLE/LE were lower as age increased in both males and females. However, their HLE/LE ratio was lower. At age 65, among male older adults, HLE was 12.02 (95% CI: 11.37–12.90) and 13.16 years (95% CI: 12.27–14.31) for the low- and high-diversity groups, respectively. Among female older adults, HLE was 14.74 (95% CI: 13.96–15.42) and 15.40 years (95% CI: 14.03–15.76) for the low- and high-diversity groups, respectively. Sex-stratified analyses showed that, among males, the high dietary diversity group had higher HLE and LE than the low dietary diversity group. Meanwhile, the differences in HLE/LE were relatively small. The differences in HLE between the high and low dietary diversity groups were statistically significant at the ages of 65–88 years (*p* < 0.05). Among females, although the high-diversity group exhibited higher HLE and LE, the HLE/LE ratio was higher in the low-diversity group. However, Z-tests showed that the difference in HLE between the two dietary diversity groups were statistically significant only at ages 78–79, 81–83, and 85–87 years (*p* < 0.05). These within-sex comparisons should be interpreted descriptively and do not constitute formal tests of interaction by sex.

**Table 3:** LE, HLE, and HLE/LE by dietary diversity and sex in older adults.

Age	Male							Female						
	Low dietary diversity			High dietary diversity				Low dietary diversity			High dietary diversity			
	LE	HLE	HLE/LE%	LE	HLE	HLE/LE%	Z	LE	HLE	HLE/LE%	LE	HLE	HLE/LE%	Z
65	13.00 (12.20–13.94)	12.02 (11.37–12.90)	92.46	14.22 (13.48–14.86)	13.16 (12.27–14.31)^**^	92.52	2.30	16.30 (15.18–16.96)	14.74 (13.96–15.42)	90.41	17.75 (16.86–18.23)	15.40 (14.03–15.76)	86.76	1.33
66	12.46 (11.79–13.40)	11.48 (10.94–12.33)	92.13	13.68 (12.96–14.32)	12.61 (11.82–13.70)^**^	92.18	2.48	15.63 (14.47–16.21)	14.05 (13.31–14.66)	89.88	17.01 (16.13–17.42)	14.67 (13.32–15.05)	86.28	1.36
67	12.02 (11.38–12.89)	11.02 (10.52–11.74)	91.70	13.18 (12.53–13.76)	12.10 (11.30–13.14)^***^	91.79	2.61	14.91 (13.90–15.48)	13.34 (12.64–13.93)	89.48	16.29 (15.47–16.68)	13.95 (12.70–14.33)	85.66	1.45
68	11.49 (10.96–12.35)	10.49 (10.11–11.22)	91.28	12.63 (12.10–13.25)	11.55 (10.82–12.54)^***^	91.45	2.73	14.26 (13.33–14.76)	12.71 (12.05–13.27)	89.15	15.61 (14.87–15.99)	13.28 (12.11–13.67)	85.08	1.43
69	11.05 (10.57–11.87)	10.06 (9.70–10.69)	90.98	12.14 (11.66–12.68)	11.05 (10.32–12.01)^***^	91.02	2.78	13.59 (12.76–14.09)	12.04 (11.45–12.59)	88.62	14.91 (14.18–15.32)	12.59 (11.50–12.96)	84.44	1.48
70	10.60 (10.16–11.40)	9.59 (9.27–10.20)	90.44	11.68 (11.25–12.19)	10.58 (9.82–11.44)^***^	90.57	2.96	12.96 (12.22–13.44)	11.40 (10.87–11.96)	87.98	14.26 (13.60–14.57)	11.95 (10.94–12.40)	83.80	1.60
71	10.15 (9.79–10.93)	9.14 (8.89–9.70)	90.04	11.21 (10.92–11.69)	10.11 (9.42–10.95)^***^	90.16	3.09	12.36 (11.62–12.82)	10.80 (10.29–11.26)	87.40	13.60 (12.97–13.88)	11.31 (10.38–11.76)	83.11	1.63
72	9.73 (9.37–10.50)	8.71 (8.46–9.20)	89.54	10.75 (10.44–11.25)	9.65 (8.91–10.39)^***^	89.74	3.15	11.76 (11.11–12.17)	10.21 (9.73–10.69)	86.81	12.93 (12.42–13.26)	10.65 (9.84–11.16)	82.38	1.47
73	9.31 (9.02–10.08)	8.28 (8.07–8.77)	88.95	10.30 (10.11–10.81)	9.19 (8.53–9.93)^***^	89.18	3.19	11.17 (10.63–11.58)	9.64 (9.20–10.08)	86.31	12.39 (11.86–12.63)	10.11 (9.32–10.56)	81.60	1.70
74	8.91 (8.72–9.62)	7.88 (7.69–8.32)	88.46	9.88 (9.76–10.35)	8.76 (8.10–9.41)^***^	88.61	3.54	10.63 (10.15–11.04)	9.07 (8.72–9.55)	85.31	11.76 (11.33–12.01)	9.50 (8.80–9.99)	80.84	1.69
75	8.50 (8.36–9.24)	7.46 (7.31–7.97)	87.77	9.47 (9.41–9.94)	8.33 (7.69–8.99)^***^	87.95	3.67	10.06 (9.66–10.51)	8.52 (8.22–8.96)	84.68	11.25 (10.81–11.43)	8.98 (8.37–9.49)	79.85	1.95
76	8.13 (7.96–8.85)	7.09 (6.90–7.57)	87.22	9.07 (9.04–9.54)	7.93 (7.31–8.51)^***^	87.35	3.55	9.57 (9.19–9.94)	8.05 (7.76–8.43)	84.07	10.67 (10.26–10.84)	8.43 (7.85–8.96)	79.00	1.77
77	7.81 (7.64–8.45)	6.76 (6.55–7.20)	86.53	8.68 (8.63–9.12)	7.53 (6.92–8.07)^***^	86.78	3.44	9.07 (8.76–9.42)	7.54 (7.34–7.90)	83.12	10.12 (9.81–10.36)	7.88 (7.46–8.51)^*^	77.84	1.68
78	7.44 (7.30–8.08)	6.39 (6.20–6.84)	85.89	8.31 (8.23–8.75)	7.14 (6.58–7.66)^***^	85.96	3.38	8.58 (8.34–8.95)	7.05 (6.89–7.42)	82.16	9.67 (9.34–9.79)	7.43 (6.99–8.00)^**^	76.88	2.11
79	7.11 (6.97–7.75)	6.06 (5.85–6.51)	85.15	7.93 (7.85–8.39)	6.77 (6.20–7.26)^***^	85.35	3.22	8.15 (7.91–8.44)	6.61 (6.48–6.96)	81.13	9.19 (8.90–9.30)	6.97 (6.55–7.53)^**^	75.92	2.14
80	6.83 (6.65–7.38)	5.75 (5.52–6.16)	84.30	7.62 (7.49–8.01)	6.44 (5.89–6.89)^***^	84.49	3.12	7.73 (7.48–8.03)	6.20 (6.06–6.53)	80.19	8.69 (8.41–8.83)	6.50 (6.13–7.08)^*^	74.83	1.88
81	6.53 (6.39–7.11)	5.44 (5.22–5.89)	83.35	7.28 (7.15–7.68)	6.09 (5.54–6.55)^***^	83.69	3.03	7.30 (7.09–7.62)	5.79 (5.68–6.11)	79.35	8.26 (7.99–8.39)	6.08 (5.72–6.69)^**^	73.65	1.97
82	6.26 (6.13–6.75)	5.15 (4.90–5.55)	82.29	6.94 (6.82–7.35)	5.74 (5.24–6.23)***	82.83	2.81	6.89 (6.65–7.20)	5.37 (5.28–5.68)	77.95	7.82 (7.56–7.97)	5.65 (5.32–6.28)**	72.20	2.08
83	5.97 (5.82–6.47)	4.86 (4.60–5.28)	81.43	6.65 (6.50–7.03)	5.44 (4.95–5.89)***	81.80	2.70	6.52 (6.27–6.83)	5.00 (4.91–5.32)	76.75	7.42 (7.14–7.58)	5.27 (4.93–5.87)**	71.01	2.13
84	5.69 (5.58–6.17)	4.57 (4.29–5.01)	80.45	6.38 (6.19–6.78)	5.15 (4.64–5.63)***	80.78	2.61	6.17 (5.88–6.47)	4.66 (4.53–4.97)	75.61	7.02 (6.73–7.21)	4.88 (4.57–5.51)*	69.55	1.71
85	5.44 (5.34–5.88)	4.32 (4.00–4.72)	79.35	6.09 (5.90–6.54)	4.86 (4.39–5.32)**	79.71	2.49	5.82 (5.54–6.16)	4.32 (4.22–4.61)	74.10	6.69 (6.41–6.86)	4.55 (4.24–5.16)**	68.05	2.06
86	5.21 (5.11–5.64)	4.07 (3.72–4.48)	78.19	5.85 (5.63–6.32)	4.60 (4.12–5.04)**	78.62	2.35	5.49 (5.20–5.81)	3.98 (3.89–4.28)	72.52	6.33 (6.04–6.52)	4.23 (3.91–4.81)**	66.81	2.25
87	4.97 (4.90–5.37)	3.82 (3.47–4.23)	76.81	5.56 (5.35–6.13)	4.31 (3.85–4.79)**	77.42	2.15	5.20 (4.88–5.56)	3.68 (3.60–3.96)	70.75	6.01 (5.73–6.22)	3.90 (3.61–4.50)**	64.97	2.02
88	4.75 (4.70–5.15)	3.59 (3.24–4.02)	75.62	5.34 (5.10–5.91)	4.07 (3.60–4.51)**	76.20	2.08	4.92 (4.59–5.26)	3.41 (3.32–3.67)	69.34	5.70 (5.41–5.91)	3.61 (3.31–4.19)*	63.34	1.87
89	4.55 (4.49–4.93)	3.37 (3.00–3.78)	74.15	5.09 (4.88–5.71)	3.80 (3.38–4.28)*	74.82	1.89	4.67 (4.33–5.01)	3.16 (3.06–3.39)	67.60	5.40 (5.09–5.64)	3.32 (3.03–3.93)	61.51	1.50
90	4.35 (4.28–4.73)	3.17 (2.78–3.57)	72.80	4.87 (4.66–5.51)	3.57 (3.16–4.04)*	73.41	1.78	4.41 (4.07–4.76)	2.89 (2.81–3.13)	65.54	5.13 (4.84–5.36)	3.06 (2.77–3.63)	59.69	1.61
91	4.18 (4.08–4.58)	2.98 (2.58–3.38)	71.18	4.67 (4.43–5.35)	3.37 (2.93–3.81)*	72.07	1.71	4.19 (3.84–4.53)	2.67 (2.58–2.90)	63.73	4.88 (4.58–5.12)	2.82 (2.53–3.38)	57.72	1.38
92	3.98 (3.89–4.44)	2.77 (2.39–3.18)	69.62	4.48 (4.24–5.18)	3.17 (2.75–3.64)*	70.66	1.73	3.98 (3.63–4.32)	2.45 (2.37–2.67)	61.48	4.65 (4.32–4.88)	2.59 (2.30–3.15)	55.73	1.41
93	3.83 (3.73–4.29)	2.60 (2.19–2.99)	67.93	4.28 (4.06–5.04)	2.95 (2.56–3.43)	68.81	1.52	3.77 (3.43–4.14)	2.23 (2.17–2.48)	59.19	4.41 (4.10–4.68)	2.36 (2.09–2.90)	53.45	1.16
94	3.68 (3.56–4.16)	2.44 (2.01–2.83)	66.20	4.11 (3.89–4.90)	2.77 (2.36–3.24)	67.24	1.43	3.59 (3.23–3.97)	2.05 (1.98–2.30)	57.24	4.20 (3.88–4.49)	2.15 (1.89–2.70)	51.16	0.84
95	3.51 (3.41–4.02)	2.25 (1.85–2.66)	64.16	3.94 (3.72–4.76)	2.59 (2.17–3.05)	65.61	1.44	3.42 (3.06–3.83)	1.87 (1.81–2.14)	54.78	4.02 (3.68–4.31)	1.97 (1.70–2.50)	49.05	0.86
96	3.38 (3.26–3.89)	2.11 (1.70–2.49)	62.42	3.79 (3.57–4.63)	2.41 (2.00–2.87)	63.67	1.34	3.25 (2.90–3.71)	1.70 (1.63–1.97)	52.19	3.84 (3.50–4.13)	1.80 (1.53–2.31)	46.84	0.86
97	3.25 (3.13–3.78)	1.97 (1.55–2.35)	60.52	3.65 (3.43–4.49)	2.25 (1.84–2.71)	61.75	1.28	3.11 (2.75–3.59)	1.55 (1.49–1.83)	49.96	3.66 (3.34–3.98)	1.64 (1.38–2.14)	44.65	0.73
98	3.12 (3.01–3.68)	1.83 (1.42–2.20)	58.51	3.50 (3.29–4.41)	2.09 (1.69–2.56)	59.78	1.22	2.97 (2.63–3.47)	1.41 (1.36–1.69)	47.62	3.50 (3.18–3.84)	1.49 (1.24–1.96)	42.49	0.63
99	3.02 (2.89–3.57)	1.71 (1.29–2.07)	56.52	3.37 (3.18–4.28)	1.95 (1.54–2.40)	57.84	1.13	2.85 (2.50–3.38)	1.28 (1.23–1.56)	45.13	3.36 (3.02–3.71)	1.34 (1.11–1.82)	40.08	0.53
100	2.90 (2.77–3.48)	1.58 (1.18–1.95)	54.30	3.25 (3.04–4.20)	1.81 (1.41–2.27)	55.88	1.12	2.73 (2.39–3.29)	1.16 (1.10–1.45)	42.56	3.22 (2.88–3.60)	1.22 (0.99–1.67)	37.92	0.52

Values in parentheses are 95% confidence intervals (CIs). ^***^p < 0.001, ^**^p < 0.05, ^*^p < 0.1.

### 3.5 Urban-rural differences in HLE by dietary diversity among older adults

As shown in Table 4, both LE and HLE were higher in the high dietary diversity group than in the low dietary diversity group in both urban and rural areas. However, the HLE/LE ratio was higher in the low dietary diversity group than in the high dietary diversity group in urban areas, whereas the two groups had similar HLE/LE ratios in rural areas. Meanwhile, both groups had similar HLE/LE ratios in rural areas. Among urban older adults aged 65 years, HLE was 14.24 (95% CI: 13.96–15.57) and 15.86 years (95% CI: 15.00–16.00) for the low- and high-diversity groups, respectively. Among rural older adults, HLE was 12.40 (95% CI: 11.57–13.10) and 12.68 years (95% CI: 12.21–13.79) for the low- and high-diversity groups, respectively. Among urban older adults, the high dietary diversity group had higher LE and HLE at all ages, but a lower HLE/LE ratio, with statistically significant differences in HLE between the high and low dietary diversity groups (*p* < 0.05). However, among rural older adults, no substantial differences were observed between the two groups in LE, HLE, or the HLE/LE ratio. Moreover, none of the between-group differences in HLE were statistically significant at any age (*p* > 0.05). These within-residence comparisons should be interpreted descriptively and do not constitute formal tests of interaction by residence.

**Table 4:** LE, HLE, and HLE/LE by dietary diversity and urban–rural in older adults.

Age	Urban							Rural						
	Low dietary diversity			High dietary diversity				Low dietary diversity			High dietary diversity			
	LE	HLE	HLE/LE%	LE	HLE	HLE/LE%	Z	LE	HLE	HLE/LE%	LE	HLE	HLE/LE%	Z
65	15.51 (14.63–16.94)	14.24 (13.96–15.57)	91.79	17.79 (15.76–19.99)	15.86 (15.00–16.00)^**^	89.14	2.15	13.66 (12.68–14.91)	12.40 (11.57–13.10)	90.80	14.01 (12.81–15.35)	12.68 (12.21–13.79)	90.52	0.33
66	14.92 (14.09–16.3)	13.62 (13.37–14.90)	91.30	17.16 (15.08–19.26)	15.17 (14.34–15.33)^**^	88.38	2.16	13.09 (12.14–14.28)	11.86 (11.05–12.47)	90.65	13.45 (12.25–14.72)	12.13 (11.70–13.19)	90.17	0.34
67	14.23 (13.55–15.59)	12.94 (12.78–14.21)	90.96	16.45 (14.55–18.55)	14.46 (13.74–14.63)^**^	87.90	2.27	12.48 (11.68–13.60)	11.27 (10.60–11.88)	90.30	12.85 (11.70–14.15)	11.54 (11.22–12.63)	89.79	0.38
68	13.67 (13.0–14.96)	12.35 (12.21–13.57)	90.36	15.81 (13.94–17.89)	13.82 (13.16–14.01)^**^	87.38	2.29	11.94 (11.16–13.00)	10.73 (10.08–11.31)	89.86	12.28 (11.12–13.61)	10.98 (10.69–12.00)	89.36	0.38
69	13.11 (12.49–14.35)	11.77 (11.64–12.93)	89.75	15.17 (13.40–17.20)	13.15 (12.53–13.39)^**^	86.73	2.29	11.43 (10.69–12.38)	10.24 (9.62–10.71)	89.57	11.78 (10.65–12.99)	10.50 (10.22–11.46)	89.17	0.44
70	12.54 (11.95–13.77)	11.18 (11.04–12.34)	89.14	14.56 (12.79–16.54)	12.50 (11.92–12.75)^**^	85.88	2.17	10.93 (10.23–11.80)	9.75 (9.19–10.11)	89.19	11.23 (10.11–12.43)	9.98 (9.75–10.96)	88.83	0.41
71	11.97 (11.45–13.17)	10.61 (10.53–11.71)	88.65	13.94 (12.28–15.92)	11.87 (11.37–12.19)^**^	85.11	2.19	10.41 (9.77–11.28)	9.25 (8.74–9.58)	88.84	10.76 (9.63–11.90)	9.52 (9.32–10.38)	88.48	0.55
72	11.42 (10.95–12.57)	10.02 (10.03–11.08)	87.77	13.39 (11.75–15.33)	11.31 (10.77–11.59)^**^	84.44	2.42	9.93 (9.38–10.71)	8.79 (8.34–9.09)	88.54	10.24 (9.14–11.36)	9.00 (8.84–9.89)	87.90	0.46
73	10.87 (10.43–12.01)	9.47 (9.52–10.52)	87.15	12.8 (11.24–14.67)	10.67 (10.17–11.02)^**^	83.42	2.27	9.45 (8.97–10.19)	8.33 (7.96–8.57)	88.15	9.74 (8.73–10.88)	8.52 (8.45–9.36)	87.46	0.45
74	10.44 (9.92–11.49)	9.00 (9.03–9.98)	86.24	12.27 (10.77–14.09)	10.12 (9.67–10.45)^**^	82.43	2.22	8.97 (8.59–9.66)	7.87 (7.59–8.09)	87.77	9.31 (8.26–10.39)	8.11 (8.03–8.92)	87.14	0.62
75	9.90 (9.43–10.99)	8.46 (8.51–9.45)	85.43	11.78 (10.29–13.48)	9.58 (9.17–9.90)^**^	81.35	2.25	8.56 (8.21–9.18)	7.47 (7.21–7.62)	87.31	8.90 (7.88–9.91)	7.72 (7.68–8.46)	86.74	0.69
76	9.46 (8.93–10.48)	7.99 (8.05–8.93)	84.40	11.21 (9.83–12.94)	9.01 (8.58–9.35)^**^	80.41	2.12	8.16 (7.82–8.71)	7.08 (6.83–7.22)	86.78	8.46 (7.45–9.45)	7.29 (7.25–8.04)	86.13	0.57
77	9.07 (8.47–9.99)	7.56 (7.59–8.42)	83.32	10.75 (9.40–12.41)	8.50 (8.06–8.86)^**^	79.06	1.95	7.81 (7.41–8.27)	6.72 (6.50–6.84)	86.12	8.04 (7.06–9.03)	6.89 (6.83–7.62)	85.80	0.49
78	8.63 (8.04–9.49)	7.10 (7.15–7.90)	82.18	10.27 (8.99–11.95)	7.99 (7.60–8.34)^**^	77.85	1.96	7.37 (7.05–7.84)	6.32 (6.14–6.50)	85.69	7.66 (6.69–8.57)	6.53 (6.42–7.22)	85.18	0.60
79	8.18 (7.61–9.05)	6.63 (6.71–7.45)	81.01	9.81 (8.57–11.42)	7.51 (7.14–7.91)^*^	76.51	1.86	7.03 (6.67–7.41)	5.99 (5.83–6.14)	85.24	7.28 (6.34–8.20)	6.17 (6.03–6.81)	84.71	0.53
80	7.8 (7.2–8.63)	6.21 (6.29–6.98)	79.58	9.34 (8.15–10.96)	7.02 (6.65–7.42)^*^	75.23	1.79	6.68 (6.32–7.06)	5.65 (5.51–5.81)	84.61	6.91 (6.02–7.80)	5.80 (5.65–6.48)	83.99	0.42
81	7.44 (6.82–8.21)	5.82 (5.89–6.54)	78.23	8.95 (7.76–10.50)	6.59 (6.20–6.98)^*^	73.63	1.70	6.35 (6.00–6.72)	5.33 (5.14–5.48)	83.97	6.57 (5.70–7.41)	5.48 (5.26–6.11)	83.43	0.44
82	7.06 (6.46–7.81)	5.41 (5.48–6.13)	76.70	8.54 (7.42–10.10)	6.15 (5.74–6.54)	72.01	1.61	6.03 (5.67–6.39)	5.03 (4.81–5.17)	83.47	6.26 (5.40–7.07)	5.19 (4.94–5.76)	82.94	0.47
83	6.71 (6.08–7.46)	5.04 (5.12–5.71)	75.13	8.15 (7.06–9.63)	5.72 (5.31–6.10)	70.25	1.59	5.74 (5.37–6.08)	4.75 (4.50–4.90)	82.81	5.94 (5.12–6.73)	4.89 (4.61–5.45)	82.31	0.37
84	6.39 (5.78–7.11)	4.69 (4.75–5.38)	73.41	7.80 (6.74–9.29)	5.33 (4.91–5.71)	68.40	1.43	5.42 (5.09–5.79)	4.45 (4.23–4.62)	82.13	5.65 (4.85–6.38)	4.60 (4.31–5.14)	81.54	0.42
85	6.08 (5.46–6.78)	4.34 (4.41–4.98)	71.39	7.39 (6.40–8.89)	4.92 (4.52–5.32)	66.56	1.35	5.18 (4.80–5.53)	4.21 (3.94–4.34)	81.40	5.38 (4.59–6.07)	4.35 (4.03–4.86)	80.96	0.38
86	5.8 (5.17–6.47)	4.03 (4.05–4.65)	69.55	7.08 (6.12–8.53)	4.57 (4.16–4.96)	64.59	1.27	4.91 (4.55–5.27)	3.96 (3.69–4.08)	80.60	5.08 (4.34–5.81)	4.06 (3.78–4.58)	80.00	0.28
87	5.51 (4.90–6.20)	3.71 (3.71–4.30)	67.28	6.77 (5.85–8.24)	4.22 (3.81–4.59)	62.41	1.24	4.68 (4.29–5.03)	3.73 (3.43–3.84)	79.77	4.83 (4.13–5.50)	3.83 (3.50–4.32)	79.31	0.27
88	5.28 (4.64–5.91)	3.44 (3.40–3.99)	65.11	6.47 (5.57–7.91)	3.89 (3.49–4.25)	60.07	1.10	4.43 (4.06–4.77)	3.50 (3.20–3.63)	78.96	4.58 (3.90–5.23)	3.59 (3.26–4.04)	78.33	0.25
89	5.03 (4.40–5.66)	3.16 (3.11–3.68)	62.87	6.2 (5.33–7.61)	3.58 (3.18–3.90)	57.65	1.06	4.20 (3.84–4.55)	3.27 (3.00–3.41)	77.92	4.39 (3.70–4.97)	3.40 (3.04–3.83)	77.44	0.34
90	4.81 (4.17–5.44)	2.90 (2.83–3.40)	60.36	5.94 (5.07–7.34)	3.29 (2.88–3.60)	55.39	1.00	4.00 (3.63–4.37)	3.08 (2.79–3.20)	76.91	4.16 (3.51–4.75)	3.18 (2.81–3.60)	76.48	0.28
91	4.58 (3.95–5.22)	2.66 (2.58–3.13)	57.93	5.69 (4.85–7.08)	3.00 (2.61–3.31)	52.75	0.94	3.82 (3.44–4.16)	2.90 (2.60–3.02)	75.82	3.95 (3.33–4.51)	2.98 (2.60–3.39)	75.51	0.23
92	4.37 (3.76–5.01)	2.41 (2.33–2.87)	55.02	5.44 (4.60–6.81)	2.73 (2.37–3.03)	50.19	0.89	3.62 (3.26–3.99)	2.70 (2.42–2.83)	74.72	3.75 (3.16–4.28)	2.79 (2.42–3.18)	74.28	0.23
93	4.19 (3.57–4.82)	2.20 (2.10–2.63)	52.52	5.24 (4.41–6.64)	2.50 (2.13–2.77)	47.80	0.86	3.44 (3.08–3.81)	2.53 (2.24–2.66)	73.49	3.57 (3.00–4.08)	2.62 (2.24–3.01)	73.37	0.25
94	4.01 (3.40–4.64)	1.99 (1.90–2.40)	49.57	5.04 (4.21–6.53)	2.27 (1.90–2.52)	44.98	0.82	3.27 (2.93–3.65)	2.37 (2.08–2.50)	72.31	3.42 (2.86–3.88)	2.46 (2.08–2.80)	72.06	0.27
95	3.84 (3.23–4.48)	1.81 (1.71–2.17)	46.99	4.84 (4.04–6.46)	2.05 (1.69–2.29)	42.36	0.77	3.14 (2.80–3.48)	2.23 (1.93–2.36)	71.13	3.26 (2.72–3.69)	2.31 (1.92–2.63)	70.96	0.22
96	3.70 (3.09–4.38)	1.63 (1.52–1.97)	44.05	4.66 (3.86–6.41)	1.85 (1.50–2.06)	39.67	0.72	2.99 (2.65–3.33)	2.09 (1.78–2.21)	69.84	3.11 (2.59–3.52)	2.16 (1.78–2.46)	69.53	0.21
97	3.56 (2.95–4.30)	1.46 (1.35–1.78)	41.16	4.49 (3.71–6.33)	1.67 (1.32–1.87)	37.12	0.70	2.85 (2.53–3.21)	1.94 (1.65–2.07)	68.20	2.96 (2.48–3.39)	2.03 (1.65–2.32)	68.34	0.24
98	3.44 (2.81–4.24)	1.32 (1.20–1.62)	38.47	4.33 (3.55–6.31)	1.50 (1.16–1.69)	34.55	0.62	2.73 (2.42–3.09)	1.83 (1.52–1.93)	66.93	2.83 (2.36–3.26)	1.89 (1.53–2.17)	66.92	0.20
99	3.32 (2.69–4.15)	1.19 (1.06–1.44)	35.77	4.18 (3.41–6.26)	1.33 (1.02–1.51)	31.83	0.55	2.61 (2.31–2.97)	1.71 (1.41–1.82)	65.44	2.71 (2.26–3.14)	1.77 (1.40–2.05)	65.48	0.20
100	3.20 (2.58–4.09)	1.06 (0.93–1.30)	33.17	4.05 (3.25–6.25)	1.19 (0.89–1.35)	29.42	0.54	2.49 (2.21–2.86)	1.59 (1.30–1.70)	63.79	2.60 (2.17–3.04)	1.66 (1.30–1.91)	63.96	0.22

Values in parentheses are 95% confidence intervals (CIs). ^***^p < 0.001, ^**^p < 0.05, ^*^p < 0.1.

## 4 Discussion

### 4.1 Higher HLE and LE but lower HLE/LE among older adults with high dietary diversity

This study observed that older adults with high dietary diversity had higher LE and HLE across all age groups. This is consistent with studies reporting that greater dietary diversity is associated with higher LE and HLE in older population (33). The transition analyses revealed that individuals with high dietary diversity had a slightly higher probability of transitioning from health to disability than those with low dietary diversity. This may reflect the complex effects of dietary diversity on functional aging because greater dietary variety does not necessarily represent optimal dietary quality (34). Although a diverse diet generally provides a broader range of essential nutrients, it may also involve the consumption of less favorable food combinations. For example, a wider variety of foods may include excessive intake of processed foods high in fat, sugar, and sodium. Such dietary patterns have been associated with adverse metabolic outcomes and chronic diseases, which may be linked to a higher likelihood of disability (35). Nevertheless, the higher health-to-disability transition probability among individuals with high dietary diversity did not translate into lower LE or HLE. This may be because these individuals experienced lower mortality probabilities, and had greater opportunities to maintain or regain their functional capacity over time. Consistently, high dietary diversity was associated with a higher probability of transitioning from disability back to health, and lower probabilities of death from both healthy and disabled states. These findings suggest that dietary diversity may be associated with greater resilience after functional decline by improving nutritional adequacy, supporting immune function, facilitating tissue repair, and preserving physiological reserves (36, 37). In contrast, low dietary diversity may be associated with deficiencies in essential nutrients, including proteins, vitamins, and minerals, which have been linked to muscle loss, osteoporosis, and cardiovascular deterioration. These conditions may, in turn, be associated with higher mortality and a lower likelihood of transition from disability (38). Furthermore, the lower HLE/LE ratio observed among older adults with high dietary diversity should not necessarily be interpreted as an adverse outcome. Instead, it may reflect their longer LE, resulting in a greater absolute number of years lived with disability despite longer HLE (39). Conversely, older adults with low dietary diversity may have shorter LE and experience more rapid disease progression, potentially reducing the duration of disability and leading to a relatively higher HLE/LE ratio (40).

### 4.2 Differences in HLE between dietary diversity groups by sex

Next, although female older adults generally had longer LE and HLE, the differences in HLE between the high and low dietary diversity groups appeared larger among males than among females. Similarly, differences in health state transition probabilities between the high and low dietary diversity groups appeared larger among males. This is consistent with previous studies reporting larger associations between dietary diversity and health outcomes among males than among females (41). While the primary dataset used here (CLHLS) did not directly measure the underlying physiological or behavioral variables, the literature offers several plausible explanations for these descriptive differences. From a physiological and metabolic perspective, males have higher basal metabolic rates and greater muscle mass. This places greater demands on proteins, micronutrients, and antioxidants, and thus, greater sensitivity to nutritional deficiencies (42). When their nutrient intake is inadequate, males may be more susceptible to oxidative stress and metabolic disturbances, which have been associated with a higher risk of chronic diseases (43). In contrast, females tend to have lower risks of chronic disease and greater survival advantages under similar nutritional conditions. This has been attributed in part to protective mechanisms such as estrogen, which may partially mask the independent contribution of dietary diversity-related differences in HLE (44). From a social perspective, older females tend to be more attentive to dietary health, participate more frequently in home cooking, and actively seek nutritional information. Even when the overall dietary diversity is not high, they may compensate for certain nutritional inadequacies through food combinations and cooking practices (45). In contrast, older males may rely more heavily on external support for dietary structure and health management and may be more prone to nutritional imbalances when such support is lacking (46).

### 4.3 Differences in HLE between dietary diversity groups by urban-rural residence

Next, the differences in HLE between the high and low dietary diversity groups appeared larger among urban older adults than among rural older adults. Similarly, differences in health state transition probabilities between dietary diversity groups appeared larger among urban older adults. Meanwhile, these differences appeared smaller among rural older adults. A possible explanation is that older adults in urban areas generally have higher nutritional literacy, which may enable them to better manage the proportions of various foods and translate dietary diversity into more balanced dietary patterns (47). In contrast, even when rural older adults increase their food variety, they may be constrained by limited cooking methods, coarse food processing, and inadequate preservation conditions, which may limit the optimal combination and absorption of nutrients (48). Moreover, older adults in urban areas generally have better access to healthcare and more comprehensive chronic disease management. Although excessive energy intake may pose metabolic risks, these risks may be mitigated through accessible primary care, routine health screenings, and pharmacological interventions (49). However, in rural areas, the scarcity of medical resources may contribute to delayed diagnoses and less effective management of chronic conditions. This may reduce or obscure the potential advantages associated with higher dietary diversity (50). Furthermore, rural older adults typically perform strenuous physical labor well into later life, leading to greater metabolic demands and higher nutritional requirements than urban older adults of the same age (51). Under such conditions, additional nutrient intake may be directed toward meeting energy demands associated with physical activity rather than supporting tissue repair or physiological reserve. Consequently, any associations between dietary diversity and health outcomes may be less apparent among rural older adults.

## 5 Conclusion

This study revealed that older adults with high dietary diversity exhibited higher probabilities of transitioning from health to disability, and disability back to health, along with lower probabilities of transitioning to death from either state. They also exhibited higher LE and HLE values. These patterns were observed across sex and urban-rural residence groups. Descriptively, larger differences in HLE between dietary diversity groups were observed among males and urban older adults than among females and rural older adults. Collectively, these findings highlight differences in the observed associations between dietary diversity and healthy longevity across population subgroups, underscoring the need to consider sex and urban-rural differences when designing nutritional strategies for older adults.

## 6 Limitations

This study has several limitations that warrant further investigation. First, the dichotomization of DDS at 7 may have resulted in some loss of information and obscured potential nonlinear associations. Notably, this cutoff has been used in studies on Chinese older adults. Given the sample size and complexity of the microsimulation framework, we did not perform additional sensitivity analyses using alternative DDS categorizations or continuous DDS specifications. Future studies should evaluate the robustness of these findings using alternative approaches to modeling DDS. Second, although we adjusted for several demographic, socioeconomic, and health-related characteristics, residual confounding cannot be ruled out. For instance, unmeasured or imperfectly measured factors such as healthcare access, nutritional knowledge, oral health, and lifestyle behaviors may have influenced both dietary diversity and functional health. Therefore, causal inferences cannot be drawn from this study. Third, reverse causation is possible because individuals’ physical functioning and self-rated health may influence their dietary behaviors. Older adults with poor health, functional limitations, or cognitive impairment may modify their dietary habits or consume a narrower variety of foods. This can make it difficult to establish the temporal direction of the observed associations. Fourth, selective survival may have introduced bias because individuals with severe illness, substantial disability, poor nutritional status, or very low dietary diversity may have been less likely to survive to subsequent survey waves. Therefore, they may have been underrepresented in the analytic sample. Similarly, participants who were lost to follow-up or excluded may have differed systematically from those retained in the study. Such differential loss and exclusion may have affected the estimated health-state transition probabilities and HLE, potentially limiting the representativeness and generalizability of the findings. Fifth, dietary diversity was assessed using self-reported dietary information, which may have been subject to recall errors, reporting bias, and misclassification. These measurement errors may have affected the magnitude or direction of the observed associations. Sixth, the DDS used in this study primarily reflected dietary diversity. Although a higher DDS may indicate a broader range of food consumption, it does not account for differences in food quantity or the overall quality of specific dietary patterns. Future studies incorporating comprehensive dietary quality indices are needed to further explore the relationship between dietary patterns and HLE in older adults. Finally, the number of bootstrap resampling iterations was limited to 30 because of the substantial computational burden of the microsimulation model. Future studies with greater computational resources should employ more bootstrap repetitions to further validate the stability of the estimates.

## Data Availability

Publicly available datasets were analyzed in this study. This data can be found here: https://opendata.pku.edu.cn/dataverse/CHADS.
